# Crystal structure of *Vmo*Lac, a tentative quorum quenching lactonase from the extremophilic crenarchaeon *Vulcanisaeta moutnovskia*

**DOI:** 10.1038/srep08372

**Published:** 2015-02-11

**Authors:** Julien Hiblot, Janek Bzdrenga, Charlotte Champion, Eric Chabriere, Mikael Elias

**Affiliations:** 1 URMITE UMR CNRS-IRD 6236, IFR48, Faculté de Médecine et de Pharmacie, Université de la Méditerranée, Marseille, France; 2 University of Minnesota, Department of Biochemistry, Molecular Biology and Biophysics & Biotechnology Institute, St. Paul, MN 55108, USA

## Abstract

A new representative of the Phosphotriesterase-Like Lactonases (PLLs) family from the hyperthermophilic crenarchaeon *Vulcanisaeta moutnovskia* has been characterized and crystallized. *Vmo*Lac is a native, proficient lactonase with promiscuous, low phosphotriesterase activity. *Vmo*Lac therefore represents an interesting candidate for engineering studies, with the aim of developing an efficient bacterial quorum-quenching agent. Here, we provide an extensive biochemical and kinetic characterization of *Vmo*Lac and describe the X-ray structures of the enzyme bound to a fatty acid and to its cognate substrate 3-oxo-C10 AHL (Acyl-Homoserine Lactone). The structures highlight possible structural determinants that may be involved in its extreme thermal stability (Tm = 128°C). Moreover, the structure reveals that the substrate binding mode of *Vmo*Lac significantly differs from those of its close homologues, possibly explaining the substrate specificity of the enzyme. Finally, we describe the specific interactions between the enzyme and its substrate, and discuss the possible lactone hydrolysis mechanism of *Vmo*Lac.

Phosphotriesterase-Like Lactonases (PLLs) compose a class of lactonases (EC 3.1.1.25) that has long been mistaken for the organophosphorus-degrading enzymes phosphotriesterases (PTEs; EC 3.1.8.1)^1^. Indeed, several PLLs were initially isolated and characterized by virtue of their organophosphorous insecticide degrading abilities^2^,^3^,^4^. In contrast with known PTEs that hydrolyze these compounds with high efficiency^5^, PLLs are less proficient^3^,^4^. In fact, PLLs are native lactonases that are endowed with promiscuous phosphotriesterase activity^1^,^6^, and it might be the progenitors of PTEs that may have diverged from PLLs upon the first use of organophosphorous insecticides in the 1950's^1^.

The PLL family has been subdivided into two sub-classes based on their structures and catalytic preferences^1^: the PLLs-A, such as *Sso*Pox^7^, *Sis*Lac^8^ and PPH^1^ are capable of hydrolyzing with high efficiency both Acyl-Homoserine Lactones (AHLs) and oxo-lactones, whereas the PLLs-B, *e.g.,* *Dr*OPH and *Gk*L^9^,^10^, comprise exclusive oxo-lactonases. The biological function of PLLs remains unclear but some evidence (*e.g.,* the enantiospecificity of PLL-As^7^) may indicate a role of some PLLs in quorum sensing. Indeed, the ability of these enzymes to hydrolyze AHLs enables them to interfere with bacterial communication^11^, a property that may be used to develop new approaches to annihilate bacterial pathogens' virulence^12^,^13^,^14^,^15^.

PLLs belong to the amidohydrolase superfamily^16^ and exhibit a (β/α)_8_ topology containing a bi-metallic active site that is coordinated by four histidines, an aspartic acid and a carboxylated lysine^17^,^18^. The bi-metallic center acts as a Lewis acid and activates a bridging, putatively catalytic, water molecule into a hydroxide anion, which subsequently serves as a nucleophile for the organophosphorous compounds or lactone hydrolysis. The active site loops 7 and 8 mediate the substrate specificity^17^,^19^: indeed, PTEs and PLLs mainly differ by the size and conformations of these loops^20^. In particular, in the PLL *Sso*Pox, the sole loop 8 position 263 modulates the promiscuous phosphotriesterase and lactonase activities, by altering the active site loop conformational landscape^7^.

*Vmo*Lac (YP_004245953) is a recently identified enzyme that was isolated from the hyperthermophilic crenarchaeon *Vulcanisaeta moutnovskia* strain 768-28^33^. Interestingly, *Vmo*Lac shares ~50% sequence identity with PLL-A and ~30% with PLL-B representatives, and may therefore possess a unique active site configuration and substrate specificity. Here we provide a biochemical and kinetic characterization of the phosphotriesterase, esterase and lactonase activities of *Vmo*Lac. Moreover, we provide the crystal structures of *Vmo*Lac in two crystal forms, both in complex with a fatty acid, and the crystal structure of *Vmo*Lac with a bound 3-oxo C10 acyl-homoserine lactone. Together, these data allow us to propose a lactone hydrolysis mechanism for *Vmo*Lac.

## Methods

### Sequence alignment

Phylogenetic analysis of PLLs were performed using *T-coffee* server (expresso)^21^,^22^, with manual improvement. The 30 sequences used for the phylogenetic analysis are issued from a previous analysis and were subsequently updated (Table S1 & S2)^20^. The *PhyML* software^23^ was employed to generate phylogenetic tree using default parameters. The sequence alignment was drawn using *BioEdit* 7.1.3. The *ClustalW* server^24^ was used to calculate protein sequence identities.

### Protein purification

The protein was produced in *E. coli* BL21(DE_3_)-pGro7/GroEL strain (TaKaRa). Purification procedure took advantage of the protein thermostability by performing an initial heat treatment of 30 minutes at 70°C. Proteins were then loaded on a StrepTrap HP chromatography column (GE Healthcare), followed by tag removal using TEV protease^25^. Finally, a size exclusion chromatography column allowed to obtain pure protein (Superdex 75 16/60, GE Healthcare)^4^,^26^. The protein molar extinction coefficient was calculated using the *PROT-PARAM* server^27^ in order to quantify the protein concentration using a nanospectrophotometer (Nanodrop, Thermofisher Scientific, France).

### Kinetic measurements

The catalytic parameters were obtained using a microplate reader (Synergy HT, BioTek, USA) controlled by the Gen5.1 software in 96-well plates of 6.2 mm path length cell for a 200 μL reaction^8^. Kinetics were performed at 25°C. The *Graph-Pad Prism 5* software was used to obtain catalytic parameters by fitting the data to the Michaelis-Menten (MM) equation. The linear part of the MM plot was fitted to a linear regression using *Graph-Pad Prism 5* software if Vmax could not be attained.

**Figure 1:**
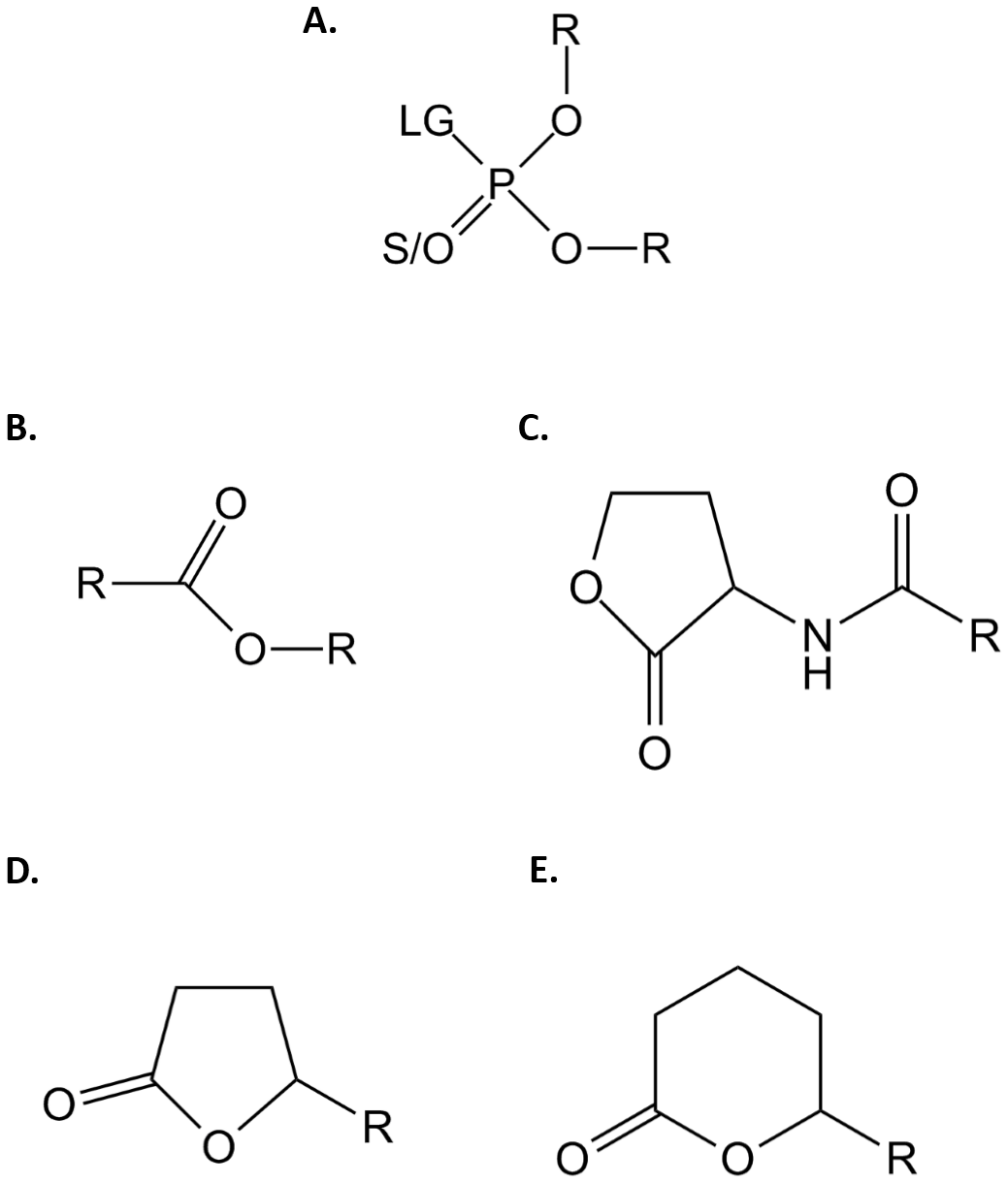
Chemical structure of the tested substrates. Chemical structures of (A.) phosphotriesters, (B.) esters, (C.) Acyl-Homoserine Lactones, (D.) γ-lactones and (E.) δ-lactones. For phosphotriesters, R corresponds to different nature of the substituents; LG corresponds to the leaving group, which can be F, S-R, O-R or CN. The terminal substituent could be S atom if the molecule is a thionophosphotriester or an O atom if the molecule is an oxonophosphotriester. For esters, R corresponds to the different nature of the substituent. For AHLs and γ/δ-lactones, R corresponds to the different size of the acyl chain.

Kinetics were performed in *activity buffer* (HEPES 50 mM pH 8, NaCl 150 mM and CoCl_2_ 0.2 mM). The time course hydrolysis of *p*NP derivatives (ε_405 nm_ = 17 000 M^−1^cm^−1^) has been measured for OPs (Fig. 1A) and esters (Fig. 1B). For malathion (Fig. S2V), 2 mM DTNB was added to the buffer (ε_412 nm_ = 13 700 M^−1^cm^−1^). The time course hydrolysis of phenyl-acetate (Fig. S2VII) and dihydrocoumarin (Fig. S2X) were monitored at 270 nm (ε_270 nm_ = 1 400 M^−1^cm^−1^) and at 412 nm for the coumarin nerve agent derivative of cyclosarin (CMP-coumarin Fig. S2VI; ε_412 nm_ = 37 000 M^−1^cm^−1^). The lactonase activities were performed in *lactonase buffer* (Bicine 2.5 mM pH 8.3, NaCl 150 mM, CoCl_2 _0.2 mM, Cresol purple 0.25 mM and DMSO 0.5%) with different AHLs (Fig. 1C) [*i.e.* C4-AHL (*r*), 1 mM; C6-AHL (r), 2 mM; 3-oxo-C6-AHL (*l*), 2 mM; C8-AHL (*r*), 1 mM; 3-oxo-C8-AHL (*l*), 2 mM; and 3-oxo-C10-AHL (*l*), 2 mM] (Fig. S2XI-XVI) and oxo-lactones (Fig. 1D E) [*i.e.,* ε-caprolactone, 5 mM; γ-heptanolide (*r*), 5 mM; nonanoic-γ-lactone (*r*), 5 mM; nonanoic-δ-lactone (*r*), 5 mM; undecanoic-γ-lactone (*r*), 5 mM; dodecanoic-γ-lactone (*r*), 5 mM] (Fig. S2XVII-XXIII). The lactone ring hydrolysis was followed by acidification of the medium for which Cresol purple serves as pH indicator (pK_a_ 8.3 at 25°C, 577 nm).

### Melting temperature (T_m_) determination

Circular Dichroïsm (CD) spectra were recorded with a Jasco J-810 spectropolarimeter equipped with a Pelletier type temperature control system (Jasco PTC-4235) both monitored by the *Spectra Manager* software. Samples were placed in 1-mm-thick quartz cell and the melting temperature of the protein was determined by following its denaturation at 222 nm in 10 mM sodium phosphate buffer at pH 7.5. Temperature was increased from 20 to 90°C (at 1°C min^−1^) and increasing concentrations of guanidinium chloride (4–6 M) were applied. A theoretical T_m_ without guanidinium chloride was then determined by extrapolating at the y-intercept by a linear fit using the *Graph-Pad Prism 5* software.

### Crystallisation

*Vmo*Lac was concentrated to 20 mg/mL for crystallization trials. Crystals were obtained in different conditions from Structure Screen 1 + 2 (Molecular Dimensions) after 2 weeks at 293 K in drops (2:1 and 1:1 protein:reservoir ratio) using the sitting drop vapor diffusion method in a 96-well plate. In addition of what was previously published (*i.e.* 400 mM ammonium dihydrogen phosphate)^28^, crystals were obtained under conditions G9 (100 mM sodium citrate pH 5.6, 10 mM ferric chloride and 10% v/v jeffamine M-600), C4 (100 mM sodium HEPES pH 7.5, 800 mM sodium and potassium dihydrogen phosphate) and E7 (100 mM Tris pH 8.5, 1.5 M ammonium sulfate and 12% v/v glycerol). The crystals from the G9 and E7 conditions had the same P6_4_ space group and the ones issued from the C4 condition had an alternative P622 group space. The crystals from E7 were soaked into the well solution supplemented by 2 mM of 3-oxo-C10 AHL for 1 min prior to flash cooling.

### Data collection, structure resolution and refinement

The crystals were transferred into a cryo-protectant solution (reservoir solution plus 20% (v/v) glycerol) before being flash cooled in liquid nitrogen. An x-ray diffraction dataset was collected at 100 K using synchrotron radiation at the ID29 beam line (ESRF, Grenoble) and a PILATUS-6M detector. X-ray diffraction data were integrated and scaled with the *XDS* package^29^ (Table 1). The phases were obtained by molecular replacement using *PHASER* and the *Sso*Pox structure as a starting model (PDB ID 2vc5)^28^. The model was subsequently built with *Coot*^30^ and refined using *REFMAC5*^31^. A total of 2 monomers (a dimer) was found per asymmetric unit in the P6_4_ space group crystals while only one monomer per asymmetric unit was observed in the P622 space group crystals. One of these dimers was highly agitated in the crystal, resulting in a poor electron density. The models and structure factors were deposited under the Protein Data Bank (PDB) codes 4RDZ, 4RE0 and 4RDY.

### Anomalous X-ray scattering data

Two datasets were collected at 1.7 and 1.8 Å resolution at energies lower (7.700 keV) and higher (7.725 keV) than the Co-K absorption edge (7.7093 keV) (Table S3).

### Structure analysis

Crystal structures of *Sso*Pox (PDB ID 2VC5 and 2VC7), *Sis*Lac (PDB ID 4G2D), *Dr*OPH (PDB ID 2ZC1) and *Gk*L (PDB ID 3OJG) were employed for structural comparisons. Illustrations, analysis and comparisons were performed with *PyMOL*, vacuum electrostatic potentials and surface representation were determined using a solvent probe of 1.4 Å radius. The dimer interface surface together with the number of hydrogen bonds and salt bridges were computed using *PISA*^32^. The root mean square deviations (RMSD) were calculated on α-carbon using the *align* command under the *PyMOL* interface.

## Results

### *Vmo*Lac is a highly thermostable PLL-A

**Figure 2:**
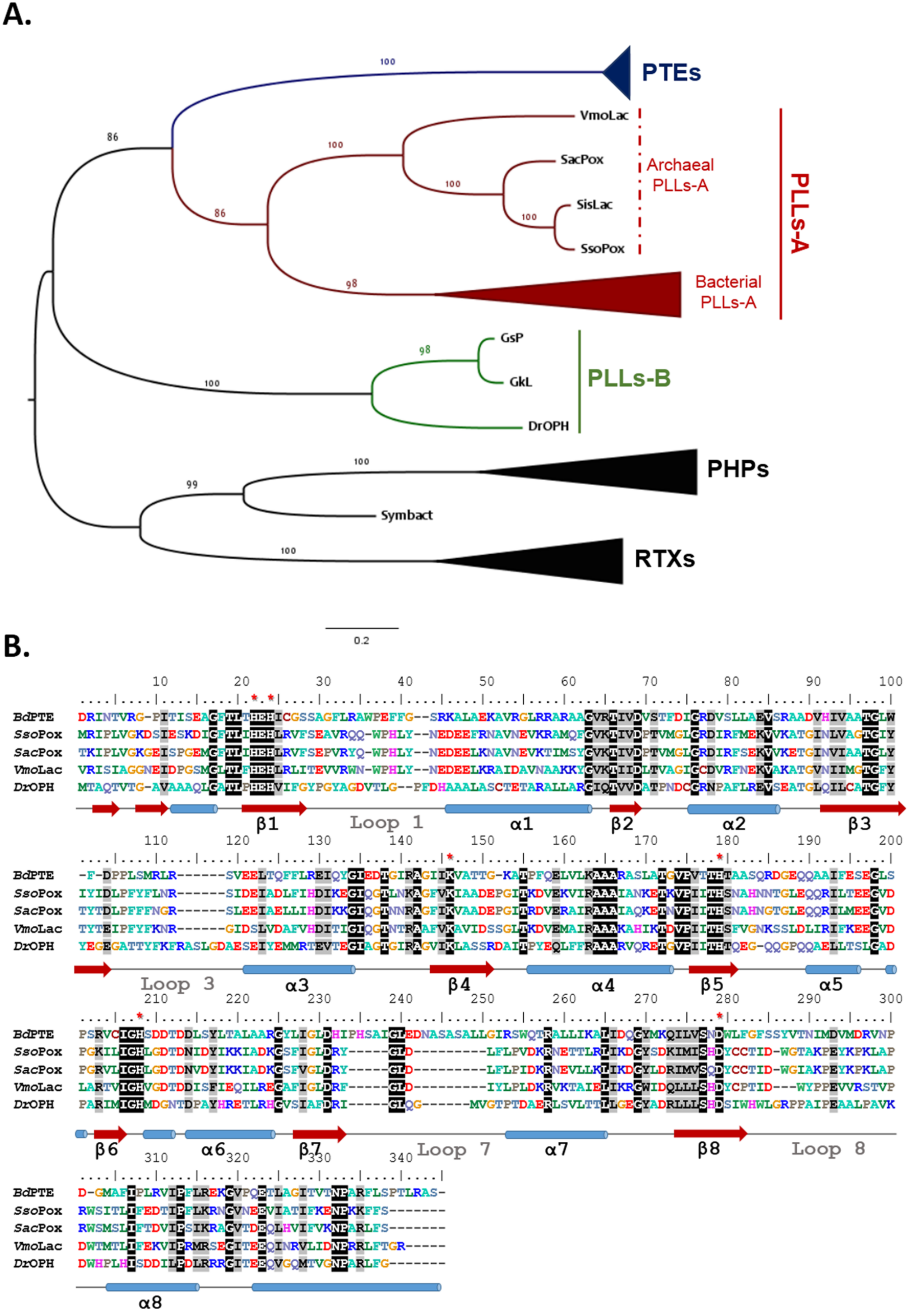
Phylogenetic analysis of PLLs. (A). Phylogenetic tree of PLL representatives and close enzymes families. Members of PLL-B are in green, while those of PLL-A are in red. For clarity, the clades with members of PTEs (blue), bacterial PLLs-A, PHP and RTX are collapsed. The sequences that were used to generate this tree are listed in supplementary Table S1. (B). Sequence alignment of *Bd*PTE from *B. diminuta*, *Sso*Pox from *S. solfataricus*, *Sac*Pox from *S. acidocaldarius*, *Vmo*Lac from *V. moutnovskia* and *Dr*OPH from *D. radiodurans*. The conserved amino acid residues are highlighted in black and similar residues in gray. The conserved active site residues that were involved in metal coordination are highlighted by red stars. The secondary structures are represented according to the *Sso*Pox structure (with red arrows depicting β-sheets and blue cylinders depicting α-helixes).

The *Vmo*Lac protein sequence was aligned with 30 sequences of PLLs, PTEs, resiniferatoxin-binding protein (RTXs) and phosphotriesterase homology proteins (PHPs) representatives (Fig. 2B, Table S1). The phylogenetic tree that was built from this sequence alignment, indicates that *Vmo*Lac belongs to the clade of the PLLs-A (Fig. 2A). However, *Vmo*Lac constitutes the most distant known representative of this clade, and shares only approximately 52% sequence identity with *Sso*Pox, 41% with PPH, < 30% with identified PLLs-B and 29% with *Bd*PTE (Table S2). The sequence alignment highlights the strict conservation of essential active site residues between *Vmo*Lac and the different clades; however, some discrepancies are visible in the regions of loops 7 and 8 of *Vmo*Lac that might account for differences in the substrate specificities. In particular, from the sequence alignment, loop 8 is shortened in *Vmo*Lac compared to his closest homologues archaeal PLLs. Moreover, a biochemical analysis of *Vmo*Lac using circular dichroïsm measurements, performed at various temperatures and guanidinium chloride concentrations, allowed us to determine the melting temperature of *Vmo*Lac: T_m_ = 128 ± 7°C (Fig. S1). This extremely high value is consistent with *Vmo*Lac originating from the extremophilic crenarchaeon *Vulcanisaeta moutnovskia* which grows between 60 and 98°C^33^, as previously concluded in works evaluating enzyme thermophilicity^34^.

### Kinetic characterization of *Vmo*Lac

*Phosphotriesterase activity*. The ability of *Vmo*Lac to hydrolyze various insecticides (*e.g.,* ethyl/methyl-paraoxon, ethyl/methyl-parathion and malathion; Fig. 1 & S2) was evaluated (Table 2). The catalytic efficiency of *Vmo*Lac against ethyl- and methyl-paraoxon is very low (k_cat_/K_M_ = 2 M^−1^.s^−1^), a much lower efficiency than that of other PLLs-A such as *Sso*Pox and *Sis*Lac (~10^2^ M^−1^.s^−1^ for ethyl-paraoxon and ~10^3^ M^−1^.s^−1^ for methyl-paraoxon)^4^,^8^. The enzyme showed no detectable activity against thiono-phosphotriester substrates (*i.e.,* ethyl/methyl-parathion and malathion). This behavior may relate to the previously described thiono-effect, in which some PLLs exhibit profound preference for oxono-phosphotriesters, whereas PTEs do not^35^. *Vmo*Lac also hydrolyzes CMP-coumarin, a cyclohexyl sarin fluorescent analogue, albeit with very low efficiency (k_cat_/K_M_ = 63.9 M^−1^.s^−1^). Overall, *Vmo*Lac is a poor phosphotriesterase. The catalytic efficiency of *Vmo*Lac for phosphotriesters is ~200-fold higher at 70°C^34^, similar to what was observed for other extremophilic archaeal PLLs^4^,^8^.

*Esterase activity*. The *Vmo*Lac kinetic parameters were recorded for several esters (*e.g.*, substrates phenyl-acetate, *p*NP-acetate and *p*NP-decanoate; Table 2, Fig. 1 & S2). This enzyme exhibits low catalytic efficiency against *p*NP-acetate (k_cat_/K_M_ = 5.48 ± 0.84 M^−1^.s^−1^) but no activity against *p*NP-decanoate. Its catalytic efficiency is ~40-fold higher at (50°C)^34^. Notably, *Vmo*Lac shows some activity against phenyl-acetate (k_cat_/K_M_ = 58.15 ± 0.95 M^−1^.s^−1^), whereas other PLL-As do not^4^,^8^.

*Lactonase activity*. The catalytic parameters of *Vmo*Lac were evaluated for a broad range of lactones, including AHLs, oxo-lactones and dihydrocoumarin (Table 2 & Fig. 1 & S2). No activity could be detected against dihydrocoumarin and AHLs with short aliphatic substituents (4 to 6 carbon atoms). However, AHLs with longer substituents are better substrates for *Vmo*Lac, including C8 and C10-AHLs (k_cat_/K_M_ = 2 × 10^3^ M^−1^.s^−1^). Oxo-lactones are the best substrates for *Vmo*Lac. Indeed, γ-caprolactone and γ-heptalactone are degraded with approximately the same catalytic efficiencies (k_cat_/K_M_ ~ 3 × 10^4^ M^−1^.s^−1^), and oxo-lactones with long aliphatic chains such as nonanoic-γ-lactone and undecanoic-δ-lactone, are hydrolyzed with high catalytic efficiencies (k_cat_/K_M_ up to ~10^6^ M^−1^.s^−1^). Intriguingly, γ and δ-dodecanoic lactone substrates have an allosteric profile of hydrolysis (K_h_ ~ 2 100 μM and 840 μM, respectively). Overall, *Vmo*Lac exhibits moderate catalytic efficiency for AHLs and shows higher rates for oxo-lactones, with a clear preference for long aliphatic chains.

### Crystal structures of *Vmo*Lac

**Figure 3:**
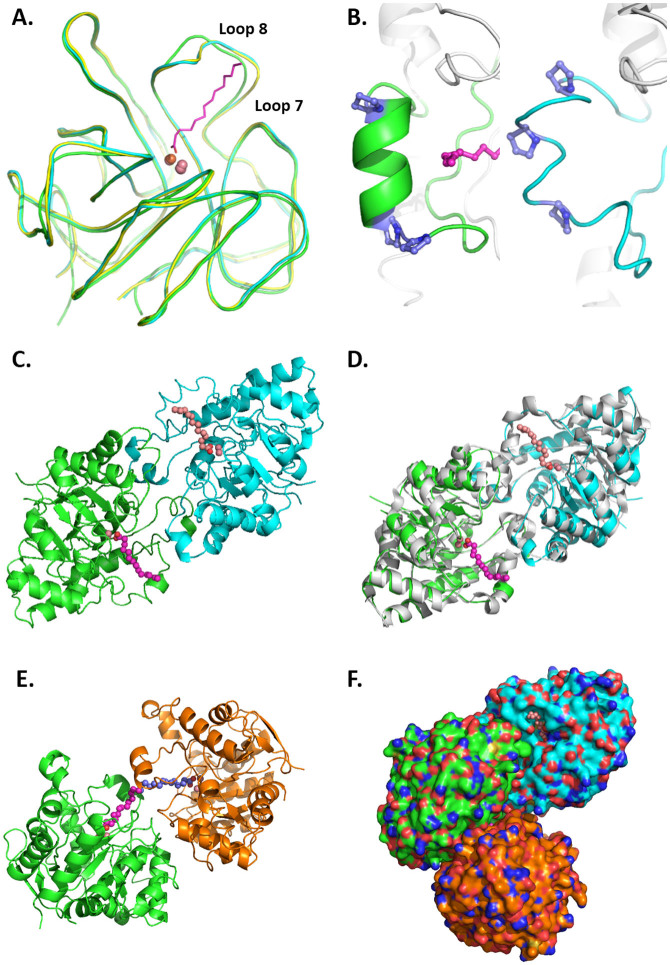
*Vmo*Lac structure comparison with that of *Sso*Pox and *Sis*Lac. (A). Structural superposition of *Vmo*Lac, *Sso*Pox (blue, 2VC5) and *Sis*Lac (yellow, 4G2D) enzymes. Iron and cobalt are represented as orange and pink spheres, respectively. Myristic acid co-crystallized with *Vmo*Lac is represented as a purple stick. (B) Loop 8 structure in *Vmo*Lac (left panel) and *Sso*Pox (right panel) context. Prolines are represented as blue sticks. The structure is in gray while loop 8 is in color, green for *Vmo*Lac and blue for *Sso*Pox. Iron and cobalt are represented as orange and pink spheres, respectively. Myristic acid co-crystallized with *Vmo*Lac is represented as a purple stick. (C). Classical dimer of *Vmo*Lac. Each monomer is colored in green and blue with respective co-crystallized myristic acid represented in light purple and pink spheres. Cobalt cations are represented as pink spheres. (D). Superposition of *Vmo*Lac dimer as in C. with *Sso*Pox dimer (2VC5) represented in light gray. (E). Alternative crystal packing interaction of *Vmo*Lac. Each monomer is colored in green and orange with respective co-crystallized myristic acid represented in light purple and dark purple spheres. The cobalt cations are represented as pink spheres. (F). Representation of the dimer, and the crystal contact of *Vmo*Lac with colorations as previously described.

The structure of *Vmo*Lac is a homodimer with the overall dimensions of the monomers being approximately 42 × 47 × 56 Å, consistent with previous observations in solution^34^. As expected, *Vmo*Lac is roughly globular and exhibits a (β/α)_8_ barrel topology that is similar to that of others PLLs such as *Sso*Pox^17^, *Sis*Lac^8^, *Dr*OPH^3^, *Gs*P^10^ and *Gk*L^36^ (Fig. S3). Indeed, the overall structure is very similar to the structure of *Sso*Pox (root-mean-square deviation (r.m.s.d.) for α-carbon atoms (over 314 atoms) of 0.87 Å; Fig. 3A). The most significant differences between the *Vmo*Lac structure and the *Sso*Pox and *Sis*Lac structures reside in the active site loop 8, which is shorter for *Vmo*Lac according to the sequence alignment (Fig. 2B). Indeed, loop 8 in the *Vmo*Lac structure is well structured into an α-helix, whereas this secondary structure is broken by proline residues in *Sso*Pox and *Sis*Lac (Fig. 3B). Remarkably, loop 8 carries the same number of proline residues (3) in all three of the counterparts, but their distribution is different: while being distributed over loop 8 in *Sso*Pox and *Sis*Lac, the 3 proline residues are located at the start and end of loop 8 in *Vmo*Lac, enabling a structure in α-helix (Fig. 3B).

*Salt bridge network analysis*. *Vmo*Lac is a highly charged protein: 43 (Asp + Glu) and 38 (Lys +Arg), for a total of 315 residues, most of them located at the protein surface. About 2/3 of these residues (54) are involved in salt bridges: *Vmo*Lac contains 40 salt bridges per monomer compared to 36 salt bridges per monomer for *Sso*Pox (using a cut-off distance of 6.5 Å). Electrostatic potential calculation suggests that one face of the protein, the active site side, is essentially negatively charged, whereas the opposite face contains positively charged clusters (Fig. S4). The protein may therefore possess a dipolar moment. One region of the enzyme is little electrostatically charged: the active site, hydrophobic channel. The salt bridges are uniformly localized on the protein surface, and most of them are involved in complex networks of interactions (Fig. S5), classical features for hyperthermophilic enzymes^18^.

*Dimer interface analysis*. The protein dimer interface involves 47 residues. This interface is mostly hydrophobic but also involves 17 hydrogen bonds and 15 salt bridges (Fig. 3C). The contacting area of 1 708 Å^2^ is very similar to the values that have been reported for other PLLs, *e.g.*, 1 770 Å^2^ for *Sis*Lac, 1 750 Å^2^ for *Sso*Pox, 1 728 Å^2^ for *Gk*L, 1 632 Å^2^ for *Gs*P and 1 473 Å^2^ for *Dr*OPH^3^,^8^,^10^,^17^,^36^.

Interestingly, *Vmo*Lac was crystallized in two different space groups. Both structures are homodimeric. The structure that was solved in P6_4 _reveals a homodimer in the asymmetric unit, as previously observed for other PLLs, such as *Sso*Pox (Fig. 3D). The other structure, solved in P622, contains only one protein molecule in the asymmetric unit. The dimer can be reconstructed by symmetry, and the observation of the other neighboring molecules reveals another, weaker interaction within the crystal packing (Fig. 3E & 3F). The contacting surface in this interaction is much smaller (616 Å^2^) and involves 4 hydrogen bonds and 4 salt bridges. The interface also involves numerous contacts *via* water molecules (19). Intriguingly, this contact mode between the two protein molecules connects the two active sites hydrophobic channels (Fig. 3E; Fig. S6).

*Comparison of the VmoLac structures*. As expected, both of the structures are nearly identical (r.m.s.d for α-carbon atoms (over 315 atoms) of 0.34 Å), but some slight differences can be observed in the active site region (loops 7 and 8; Fig. S7). In particular, loop 8 residues Y265, V269, V270 and T273 occupy slightly altered conformations, whereas loop 7 undergoes a larger conformational displacement. The largest rearrangement involves Y230, whose conformation differs by 2.2 Å between the structures. This is consistent with a higher mobility of active site loops 7 and 8, as compared to the rest of the structure.

### *Vmo*Lac is a bi-cobalt metalloenzyme

The two metal cations are coordinated by four histidines (23, 25, 171 & 200), an aspartic acid (257) and a carboxylated lysine residue (238). Both of the metal cations are bridged by a putatively catalytic water molecule. The chemical nature of the bound metal cations was investigated by X-ray anomalous scattering at the Co-K edge (1.6049 Å) and below (1.6101 Å) (Table S3). The presence of two peaks for each metal in the Bijvoet difference Fourier maps at the Co-K edge (Fig. S8) and their drop to nearly no signal under the K edge unambiguously indicate that *Vmo*Lac possesses a bi-cobalt active site.

### Active site of *Vmo*Lac

**Figure 4:**
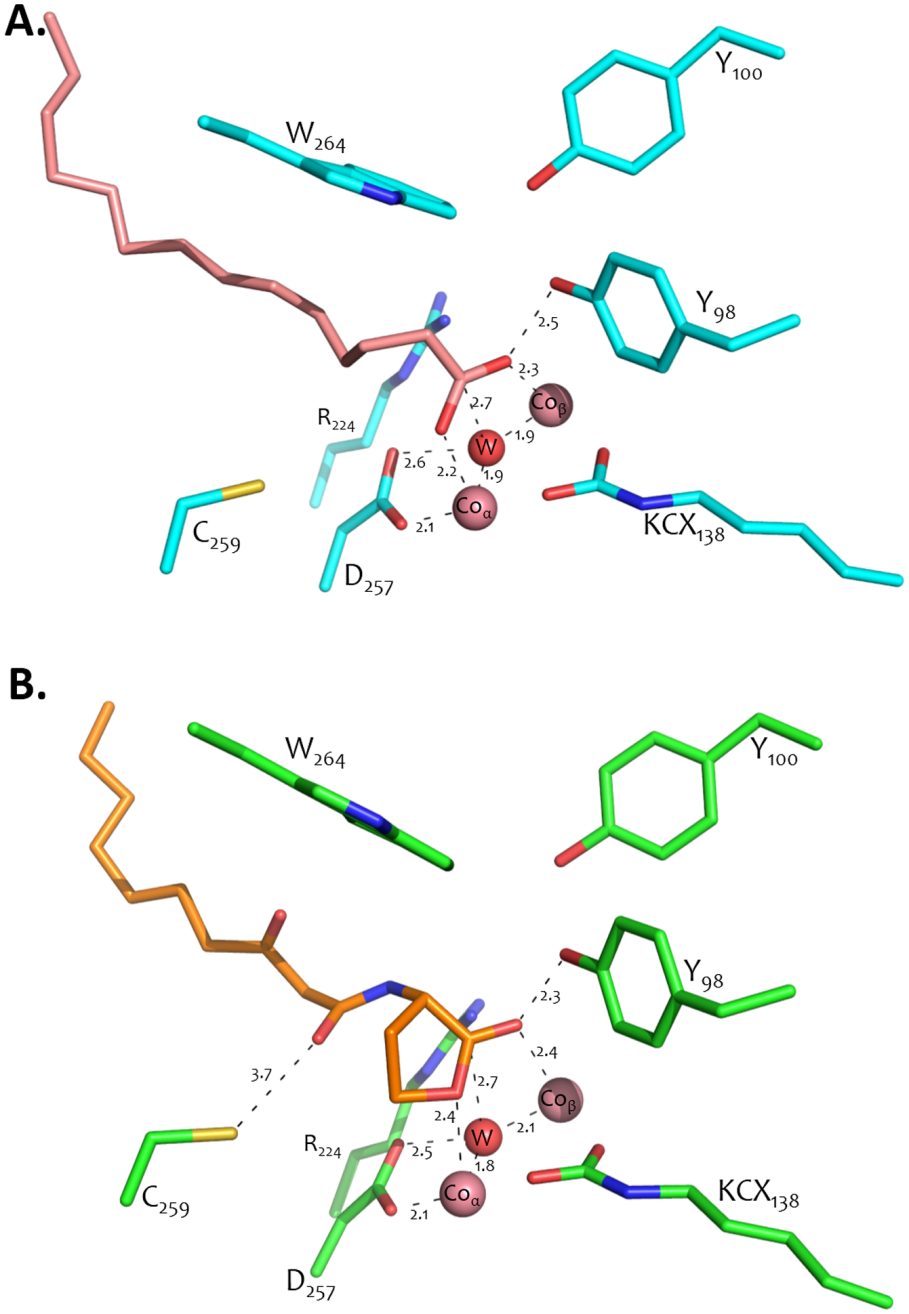
*Vmo*Lac active site ligand binding. (A). Active site residue representation of *Vmo*Lac dimer structure. The residues are represented as blue sticks. The myristic acid in the active site is represented by light pink sticks. The cobalt cations and the water molecule are represented by light pink and red spheres, respectively. (B). Active site residue representation of the complex *Vmo*Lac structure with 3-oxo-C10 AHL. The residues are represented as green sticks. The AHL is represented as orange sticks; the cobalt cations and the water molecule are represented by light pink and red spheres, respectively.

A clear extra density was observed in the active sites of both of the *Vmo*Lac structures (Fig. S9B), although no potential substrates were present in the crystallization conditions. Because of a relatively good resolution of the structures (1.8 and 2.35 Å), this unexpected density could be attributed to a fatty acid with good confidence. However, the precise nature of this fatty acid is uncertain: the number of carbon atoms is clear from the electronic density maps (14), but possible unsaturation is difficult to interpret from density maps. Therefore, this density was attributed to the simplest fatty acid that fit the observed density and that was present in the expression host *E. coli*: myristic acid. The carboxylate of the fatty acid molecule is tightly bound to the bi-metallic active site (2.2 Å_α_–2.3 Å_β_), while the long aliphatic fatty chain that sits in the hydrophobic channel is mainly formed by loop 8 residues (Fig. 4A). Notably, the bound fatty acid adopts a slightly different binding mode in both of the monomers, and movements of residues on the active site loop 7 can be observed (Ile229 and Tyr230) (Fig. S10).

### Complex with 3-oxo C10 AHL

Crystals of *Vmo*Lac were soaked with 3-oxo-C10 AHL, and the lactone sits in the hydrophobic channel of the active site (Fig. 4B & S9A & S10). The omit density map cannot be confounded with that of the fatty acid, since the bound lactone is shorter, and the lactone ring is visible in the electronic density map. Moreover, we note that both structures, solved in the same space group (P6_4_), exhibit significant conformational differences, in particular in loop 7, where Y230 undergoes a significant reorientation (1.7 Å) (Fig. S11). Noteworthy, the carboxylate group of the bound fatty acid nearly superimposes with the lactone group of the bound AHL.

**Figure 5:**
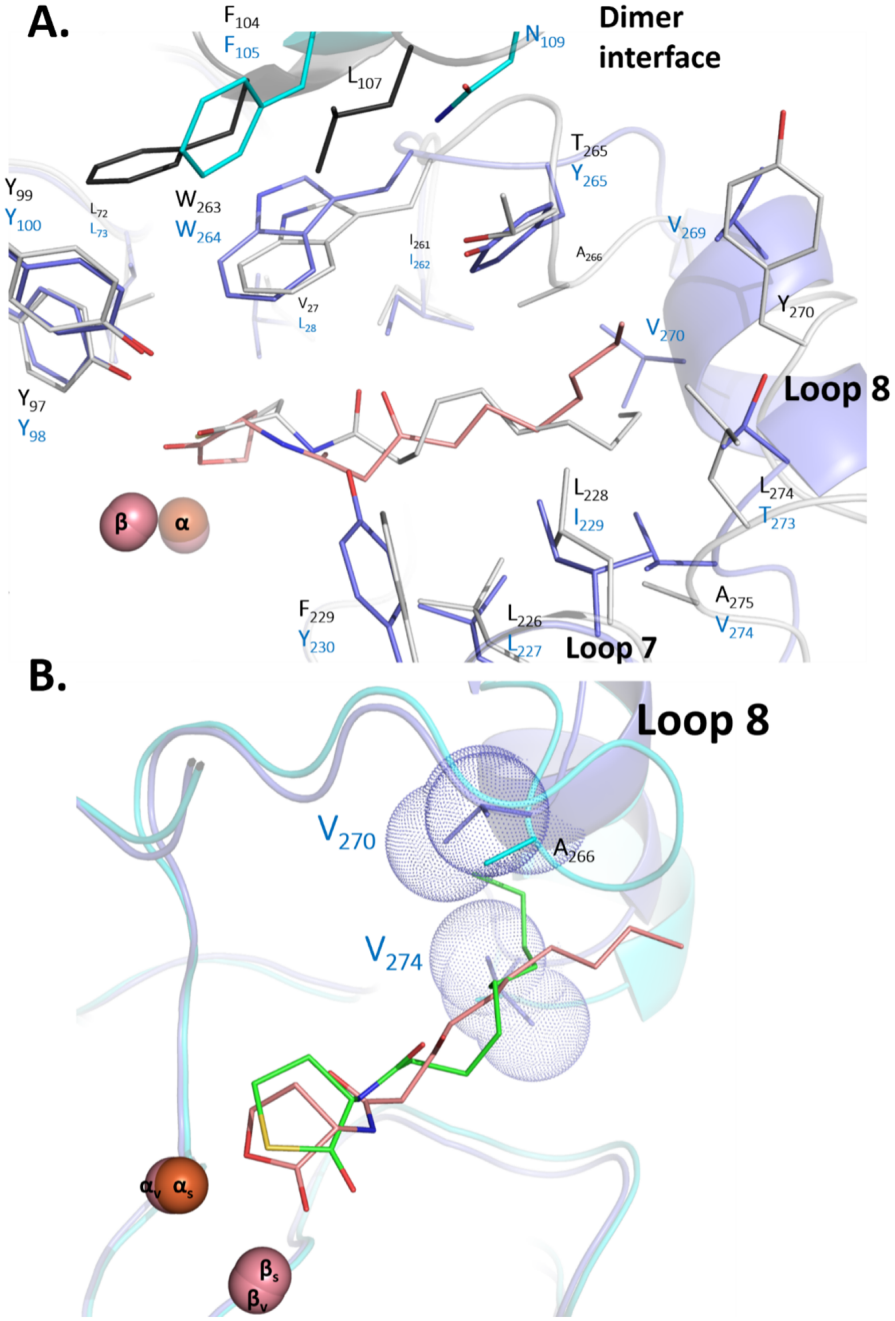
Comparison between the *Vmo*Lac and *Sso*Pox structures. (A). The *Vmo*Lac structure (in blue sticks and cartoon, residues labeled in blue) bound to 3-oxo-C10 AHL (pink sticks) was superposed onto the *Sso*Pox structure (white sticks and cartoon, residues labeled in black; PDB ID: 2vc7) bound to C10 homocysteine thiolactone (white sticks). The second monomer of the homodimer is in cyan and black, for *Vmo*Lac and *Sso*Pox, respectively. Active site metal cations are shown as spheres. (B). Zoom-in the binding of 3-oxo-C10 AHL (pink sticks; *Vmo*Lac structure) and C10 homocysteine thiolactone (green sticks; *Sso*Pox structure) into the active sites of *Vmo*Lac (violet sticks) and *Sso*Pox (cyan sticks). Active site metal cations of *Vmo*Lac (v) and *Sso*Pox (s) are shown as spheres. The van der Walls surfaces of V270 and V274 side chains are shown as blue dots.

The overall binding of the 3-oxo-C10 AHL is very similar to the previously observed binding of a lactone mimic, the inhibitor C10 homocysteine lactone in the *Sso*Pox structure^17^ (Fig. 5A & S9A). The lactone ring sits on the bi-metallic active site, while the carbonyl oxygen is bound to the β-Co (2.4 Å), and the esteric oxygen contacts the α-Co (2.4 Å). The carbonyl oxygen of the lactone ring also interacts with the Y98 O-H (2.3 Å) and the R224 guanidinium group (3.5 Å) (Fig. 4B). The 1-oxo group weakly interacts with C259 (3.7 Å), and the long aliphatic chain sits in the hydrophobic channel that is formed by loop 8. A deeper comparison highlights the fact that the lactone ring is closer to the bi-metallic center in the *Vmo*Lac structure compared to the thiolactone ring in *Sso*Pox. This discrepancy could be due to the larger van der Walls radius of sulfur compared to that of the oxygen atom, and/or by the lower polarity of sulfur compared to that of the oxygen atom. It could be also due to the significant differences found between both active sites: in particular, the substitution L28*_Vmo_*_Lac_/V27*_Sso_*_Pox_ may contribute to the observed different conformation of the bound lactone and of W264 (W263 in *Sso*Pox). The latter residue is known to be critical for substrate binding and catalytic efficiency in *Sso*Pox^37^: while W263 positions and stacks the lactone ring onto the bi-metallic active site in *Sso*Pox, it interacts very differently with the lactone ring in the *Vmo*Lac structure (Fig. 5A). Whereas several substrate binding residues are strictly conserved between both enzymes, and adopt similar conformations, such as R224*_Vmo_*_Lac_/R223*_Sso_*_Pox_, W277*_Vmo_*_Lac_/W278*_Sso_*_Pox_, Y100*_Vmo_*_Lac_/Y99*_Sso_*_Pox_, Y98*_Vmo_*_Lac_/Y97*_Sso_*_Pox_, L73*_Vmo_*_Lac_/L72*_Sso_*_Pox_, L227*_Vmo_*_Lac_/L226*_Sso_*_Pox_, I262*_Vmo_*_Lac_/I261*_Sso_*_Pox_, other residues differ and contribute to the remodelling of the binding site, such as Y230*_Vmo_*_Lac_/F229*_Sso_*_Pox_ and I229*_Vmo_*_Lac_/L228*_Sso_*_Pox_.

Moreover, the difference in the loop 8 conformation between both enzyme yield to a very different binding crevice (Fig. 5A), where very different residues are involved in the substrate binding, such as Y265*_Vmo_*_Lac_/T265*_Sso_*_Pox_, V274*_Vmo_*_Lac_/A275*_Sso_*_Pox_, T273*_Vmo_*_Lac_/L274*_Sso_*_Pox_, and also V269*_Vmo_*_Lac_, V270*_Vmo_*_Lac_, A266*_Sso_*_Pox_ and Y270*_Sso_*_Pox_. This different crevice results in the aliphatic chain binding outside loop 8 in *Vmo*Lac, whereas it binds inside loop 8 in *Sso*Pox^17^(Fig. 5B). In particular, residues V274 and V270 sterically prevents the existence of a hydrophobic channel under loop 8 in *Vmo*Lac, as opposed to the channel existing in the *Sso*Pox structure.

## Discussion

### *Vmo*Lac is a proficient lactonase

The *Vmo*Lac enzyme is a recently discovered PLL-A member from the extremophilic archaeon *V. moutnovskia* that lives between 60 and 98°C^33^,^38^. Consequently, *Vmo*Lac is highly stable and exhibits a maximum of activity at 80°C^34^. We determined that *Vmo*Lac exhibits a T_m_ value of 128 ± 7°C, a more than 20°C higher value than its hyperthermostable counterpart *Sso*Pox. The crystal structures indicate that *Vmo*Lac shares many structural determinants with its hyperthermostable PLL counterparts^18^, and other thermostable enzymes^39^, such as a very large number of surface salt bridges and a large, mainly hydrophobic homodimer interface. The structure of *Vmo*Lac also shows that the active site loop 8 is rigid and structured into an α-helix. This feature differs considerably with the closest PLLs (~50% sequence identity), where loop 8 is unstructured and floppy^8^,^37^, and might partly explain the superior thermal stability of *Vmo*Lac.

Kinetic characterization indicates that *Vmo*Lac is a proficient lactonase with promiscuous, poor esterase and phosphotriesterase activities. *Vmo*Lac clearly prefers long chain substrate, a consistent fact with the finding of a bound fatty acid (modeled as myristic acid) in the native structure. The biological role of *Vmo*Lac is uncertain: quorum quenching lactonases are found in Bacteria, Archaea and Eukarya. In many cases, these enzymes are found with no other AHL components^1^,^6^, as it seems to be for *Vmo*Lac. Therefore, the role of these enzymes in such organisms might be to interfere with quorum sensing of other organisms or to utilize AHLs as carbon and nitrogen source.

Similar to other crenarchaeal PLLs^4^,^8^,^40^, *Vmo*Lac exhibits a clear preference for oxonophosphotriesters compared to thionophosphotriesters. Notably, this preference dubbed thiono-effect^4^ is present in *Vmo*Lac despite the different nature of its bi-metallic active site: *Vmo*Lac has a bi-cobalt active site, whereas *Sso*Pox possesses an iron/cobalt bi-metallic center^17^. This observation suggests that the thiono-effect might be independent of the chemical nature of the bi-metallic center. We note that the biologically relevant metal content of *Vmo*Lac might be different, since cobalt cations were used during the enzyme purification.

*Vmo*Lac also possesses unique substrate specificity within PLLs: (i) a very low hydrolysis rates towards OPs, (ii) the ability to hydrolyze phenyl-acetate, (iii) the inability to hydrolyze dihydrocoumarin and short chain lactones and (iv) an allosteric behavior with some long chain oxo-lactones. The allosteric behaviour of *Vmo*Lac with some substrates might originate from one or a combination of the following features: the spatial proximity between active sites in the homodimer, a hypothetic alternate contact mode between monomers as seen in the crystal, or from the structural specificities of the *Vmo*Lac active site discussed below.

The structure of *Vmo*Lac indicates that the enzyme binding pocket is different from its counterparts and may account for the observed kinetics differences. A significant difference relates to L28*_Vmo_*_Lac_/V27*_Sso_*_Pox_ and W264*_Vmo_*_Lac_/W263*_Sso_*_Pox_, the latter being a key residue in *Sso*Pox that governs the active site specificity and flexibility^37^. This residue interacts differently with the bound lactone: whereas in *Sso*Pox, the indole ring of W263 makes extensive van der Walls contacts with the bound lactone ring, W264 in *Vmo*Lac interacts only weakly with the bound lactone. Given the critical role of this residue in both enzymes, being a key active site and dimer interface residue, this difference may significantly contribute to the observed differences in activity between *Vmo*Lac and its homologues.

Another major difference concerns the active site loop 8, which is involved in the substrate binding. Whereas in *Sso*Pox and *Sis*Lac^8^,^17^, loop 8 is unstructured, possibly due to 3 well-distributed proline residues along this loop, loop 8 of *Vmo*Lac forms an α-helix. This difference has major consequences on the *Vmo*Lac binding crevice: the residues V270 and V274 sterically prevents the existence of a channel under loop 8. As a result, the aliphatic acyl chain of the bound substrate interacts with the outer surface of loop 8, whereas the chain is fitting in the hydrophobic channel formed under loop 8 in *Sso*Pox^17^. This major difference in substrate binding yields different interactions between acyl chains and the enzymes: in particular, residues Y265*_Vmo_*_Lac_, V269*_Vmo_*_Lac_, T273*_Vmo_*_Lac_, I229*_Vmo_*_Lac_ are involved.

### Lactone hydrolysis mechanism

**Figure 6:**
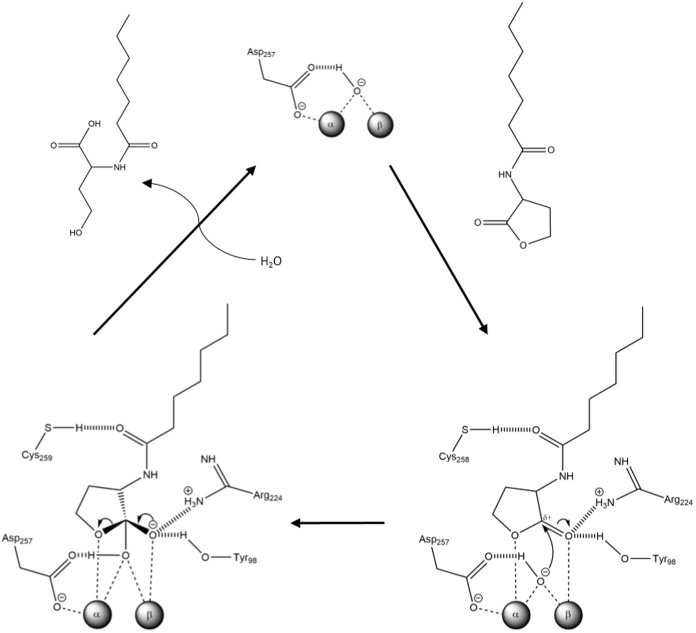
Putative catalytic mechanism of *Vmo*Lac lactonase.

The structure of *Vmo*Lac bound to its substrate 3-oxo-C10 AHL enabled us to identify the specific interactions of the enzyme with the bound lactone. The obtained complex is similar to that of *Sso*Pox bound to C10-HTL (Homoserine Thio-Lactone)^17^. We surmise here that the hydrolysis mechanism of lactones by *Vmo*Lac is therefore close to those that have been previously proposed for *Sso*Pox^17^. Indeed, the bonding of the lactone ring onto the bi-metallic center may make the lactone sp2 carbon more electrophilic and free the activated bridging water molecule. The latter may subsequently attack the lactone sp2 carbon, *via* a tetrahedral, negatively charged, intermediate that is stabilized by the β-metal. The electron pair on the oxyanion folds back, allowing the breakage of the ester bond and the formation of a carboxylic acid, and an alcoholate (Fig. 6). This alcoholate may require acidic assistance. In the homologue *Gk*L^36^,^41^, and in the other lactonase AiiA^42^,^43^, an aspartate residue (corresponding to Asp257 in *Vmo*Lac) has previously been proposed to protonate the leaving alcoholate, which remains to be clarified in *Vmo*Lac.

### The potential of *Vmo*Lac as quorum-quenching agent or an as organophosphorous compound biodecontaminant

*Vmo*Lac is an extremely thermostable enzyme, and is likely the most thermostable PLL that has been characterized thus far. Thermostability is a key feature in the use of enzymes in industrially compatible process for incorporation in chemical synthesis or other usages because thermostability is linked to easier storage, manipulation, solvent resistance and thermal resistance^39^. Due to its dual catalytic activities, *i.e*., lactonase and phosphotriesterase, *Vmo*Lac could represent an interesting target for *in vitro* evolution with the aims of developing a quorum quenching agent and an efficient organophosphorous biodecontaminant.

## Author Contributions

J.H., E.C. and M.E. designed experiments. J.H., J.B. and C.C. performed the experiments. J.H., E.C. and M.E. analysed the results. J.H., E.C. and M.E. wrote the paper. All of the authors offered a critical review of the paper.

## Supplementary Material

Supplementary information Supplementary information

## Figures and Tables

**Table 1:** Data collection and refinement statistics of *Vmo*Lac structures

Data collection			
	*Vmo*Lac-P6_4_	*Vmo*Lac-P622	VmoLac-3-oxo-C10-AHL
PDB ID	4RDZ	4RE0	4RDY
Beamline	ID29	ID29	ID29
Wavelength (Å)	0.9537	0.9537	0.9537
Detector	PILATUS 6M	PILATUS 6M	PILATUS 6M
Oscillation (°)	0.1	0.1	0.1
Number of frames	1800	1800	1200
Resolution (Å)	1.8	2.35	2.0
Space group	P6_4_	P622	P6_4_
Unit-cell parameters (Å)	a = 174.96 ; b = 174.96 ; c = 62.07 ; α = 90 ; β = 90 ; γ = 120	a = 134.67 ; b = 134.67 ; c = 126.40 ; α = 90 ; β = 90 ; γ = 120	a = 174.74 ; b = 174.74 ; c = 61.55 ; α = 90 ; β = 90 ; γ = 120
No. of observed reflections (last bin)	1 001 921(150 041)	544 316(34 394)	490 250(67 169)
No. of unique reflections(last bin)	100 625(14 960)	28 744(1 722)	72 593(9 826)
Completeness (%)(last bin)	100.0(100.0)	99.9(100.0)	99.8(99.9)
R_meas_ (%) (last bin)	7.4(63.2)	11.2(89.0)	12.2(92.1)
I/σ(I) (last bin)	25.30(4.62)	28.19(4.07)	17.94(2.89
Redundancy (last bin)	99.56(100.29)	18.94(19.97)	6.75(6.83)
CC(1/2)	99.9(91.0)	99.9(90.2)	99.8(71.2)
Refinement statistics			
Rfree/Rwork	0.16007/0.13001	0.17063/0.14090	0.17444/0.13866
No. of total model atoms	6244	2880	5844
Ramachandran favored (%)	98.94	96.86	98.43
Ramachandran outliers (%)	0.00	0.31	0.00
Generously allowed rotamers (%)	0.70	1.45	1.28
Rmsd from ideal			
Bond lengths (Å)	0.024	0.022	0.022
Bond angles (°)	2.150	2.173	2.039

**Table 2:** Enzymatic characterisation of *Vmo*Lac enzyme

	Substrates	k_cat_ (s^−1^)	K_M_ (μM)	k_cat_/K_M_ (M^−1^s^−1^)
**Phosphoesters**	**ethyl-paraoxon (I)**	(1.08 ± 0.06) × 10^−3^	581 ± 61	1.86 ± 0.22
	**methyl-paraoxon (II)**	ND	ND	2.32 ± 0.15
		1.25#	2 790#	442.58#
	**ethyl-parathion (III)**	ND	ND	ND
	**methyl-parathion (IV)**	ND	ND	ND
	**malathion (V)**	ND	ND	ND
	**CMP-coumarin (VI)**	0.13 ± 0.01	2 050 ± 257	63.90 ± 10
**Esters**	**Phenyl-acetate (VII)**	ND	ND	58.15 ± 0.95
	***p*NP-Acetate (VIII)**	(2.45 ± 0.19) × 10^−2^	4 471 ± 593	5.48 ± 0.84
		1.66#	8 190#	201.74#
	***p*NP-Decanoate (IX)**	ND	ND	ND
**Lactones**	**C8 AHL (XIV)**	0.59 ± 0.04	262 ± 54	(2.23 ± 0.48) × 10^3^
	**3-oxo C8 AHL (XV)**	0.35 ± 0.01	186 ± 37	(1.88 ± 0.38) × 10^3^
	**3-oxo C10 AHL (XVI)**	0.47 ± 0.03	231 ± 55	(2.05 ± 0.50) × 10^3^
	**γ caprolactone (*r*)**	112.30 ± 8	3709 ± 497	(3.03 ± 0.46) × 10^4^
	**γ caprolactone (*R*)**	3.04#	550#	5.56 × 10^3^#
	**γ caprolactone (*S*)**	1.89#	750#	2.53 × 10^3^#
	**γ heptalactone (XVIII)**	27.25 ± 3.1	872 ± 220	(3.12 ± 0.86) × 10^4^
	**γ nonalactone (XIX)**	44.49 ± 0.89	47 ± 11	(9.48 ± 2.18) × 10^5^
	**γ undecalactone (XX)**	8.86 ± 0.30	120 ± 22	(7.36 ± 1.37) × 10^4^
	γ dodecalactone* (XXI)	0.16 ± 0.03	2.1 ± 0.6	(7.77 ± 2.40) × 10^4^
	**δ nonalactone (XXII)**	88.91 ± 1.56	1220 ± 59	(7.29 ± 0.37) × 10^4^
	**δ undecalactone (XXIII)**	60.78 ± 2.36	105 ± 24	(5.80 ± 1.32) × 10^5^
	δ dodecalactone* (XXIV)	28.08 ± 0.44	839 ± 13	(3.35 ± 0.74) × 10^5^
	**dihydrocoumarin (X)**	ND	ND	ND

Roman numerals correspond to the related chemical structure of the substrate that is presented in Fig. S1. Kinetics were measured as triplicates, and standard deviation values are given for each parameter.

^*^substrate γ dodecalactone and δ dodecalactone showed allosteric curves with K_h_ = 2109 ± 554 μM and K_h_ = 839 ± 13 μM, respectively. The data were obtained with cobalt as cofactor. ND corresponds to not determined values because of a no or too low catalytic rate. VLH corresponds to Very Low Hydrolysis observed without the possibility of recording a value.

^#^Data from Kallnik *et al*. (2014): The lactonase activity was determined at 40°C pH 8.3 in presence of CoCl_2_, phosphotriesterase activity at 70°C pH 8 in the presence of CoCl_2_ and esterase activity at 50°C pH 6.5 in the presence of MnCl_2_. *r* corresponds to a racemic mixture of *R* and *S* enantiomers.
