# Supplementary material for: Crystal structure of VmoLac, a tentative quorum quenching lactonase from the extremophilic crenarchaeon Vulcanisaeta moutnovskia
Source: Sci Rep. 2015 Feb 11;5:8372. doi: 10.1038/srep08372 (PMC4323659; doi:10.1038/srep08372)
Supplement: Supplementary information [file srep08372-s1.pdf]

SUPPLEMENTARY INFORMATION FOR

Crystal structure of *Vmo*Lac, a tentative quorum quenching lactonase from the extremophilic crenarchaeon *Vulcanisaeta moutnovskia*

Structure of the lactonase VmoLac

Julien HIBLOT[1*], Janek BZDRENGA[2*], Charlotte CHAMPION[2], Eric CHABRIERE[2#], Mikael ELIAS[2#]

SUPPLEMENTARY INFORMATION

Figure S1: Biochemical analysis of *Vmo*Lac enzyme

Figure S2: Chemical structure of phosphoesters (I-V), esters (VI-VIII) and lactones (IX-XXIII)

Figure S3: Structural superposition of several PLL with *Vmo*Lac

Figure S4: Surface electrostatic potential of *Vmo*Lac

Figure S5: Surface salt bridge network example in the *Vmo*Lac structure

Figure S6: Contacts between *Vmo*Lac active site in the packing contact

Figure S7: Superposition of *Vmo*Lac structures solved in P64 (green) and P622 space groups.

Figure S8: Anomalous X-ray scattering characterization of the *Vmo*Lac bimetallic center

Figure S9: Fourier difference maps of the bound ligands in *Vmo*Lac structures

Figure S10: Superposition of monomers from homodimers of *Vmo*Lac bound to a fatty acid (A.) and to 3-oxo-C10 AHL (B.)

Figure S11: Superposition of the structure bound to a fatty acid with the structure bound to the AHL.

Table S1: Accession numbers of the sequences used in the phylogeny study

Table S2: Protein sequence identity between PLLs and *Bd*PTE

Table S3: Anomalous X-ray data collection

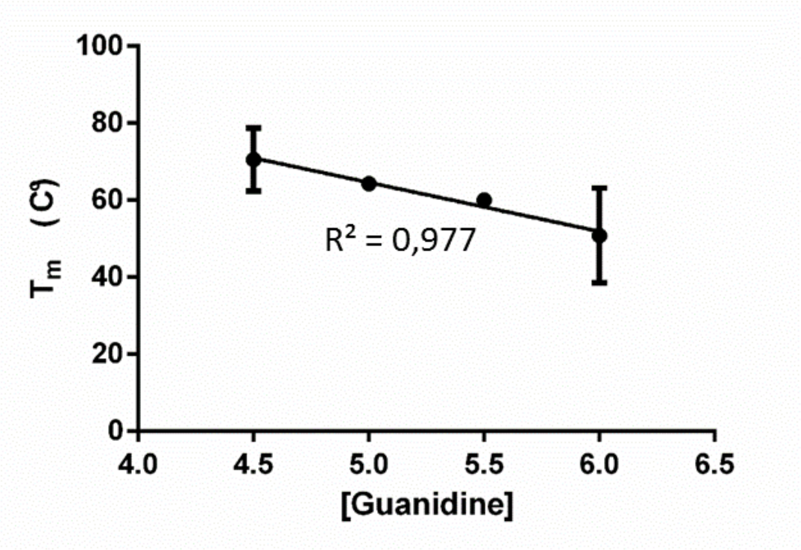

Figure S1: Biochemical analysis of the *Vmo*Lac enzyme

The protein thermostability has been evaluated using different concentration of guanidinium chloride (4.5, 5, 5.5 and 6 M). The melting-temperature (T_m) of *Vmo*Lac has been extrapolated at a guanidine concentration equal to 0 using a linear regression and was evaluated to 128 ± 7° C.

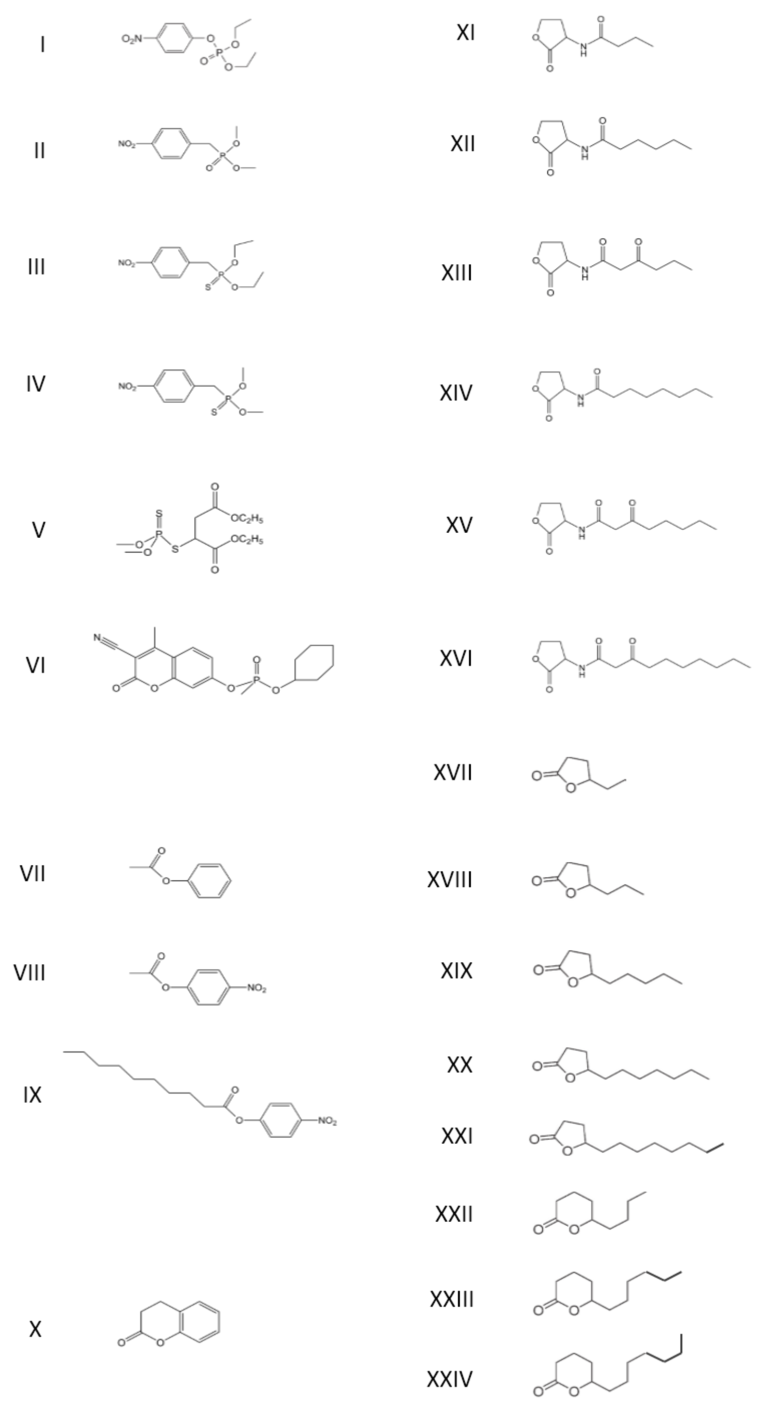

Figure S2: Chemical structure of phosphoesters (I-VI), esters (VII-IX) and lactones (X-XXIV)

Chemical structure of ethyl-paraoxon (I), methyl-paraoxon (II), ethyl-parathion (III), methyl-parathion (IV), malathion (V), CMP-Coumarin (VI) phenyl-acetate (VII), *p*NP-acetate (VIII), *p*NP-decanoate (IX), dihydrocoumarin (X), C4-AHL (XI), C6-AHL (XII), 3-oxo-C6-AHL (XIII), C8-AHL (XIV), 3-oxo-C8-AHL (XV), 3-oxo-C10-AHL (XVI), γ-caprolactone (XVII), γ-heptanolide (XVIII), Nonanoic-γ-lactone (XIX), Undecanoic-γ-lactone (XX), Dodenanoic-γ-lactone (XXI) Nonanoic-δ-lactone (XXII), Undecanoic-δ-lactone (XXIII) and Dodenanoic-δ-lactone (XXIV)

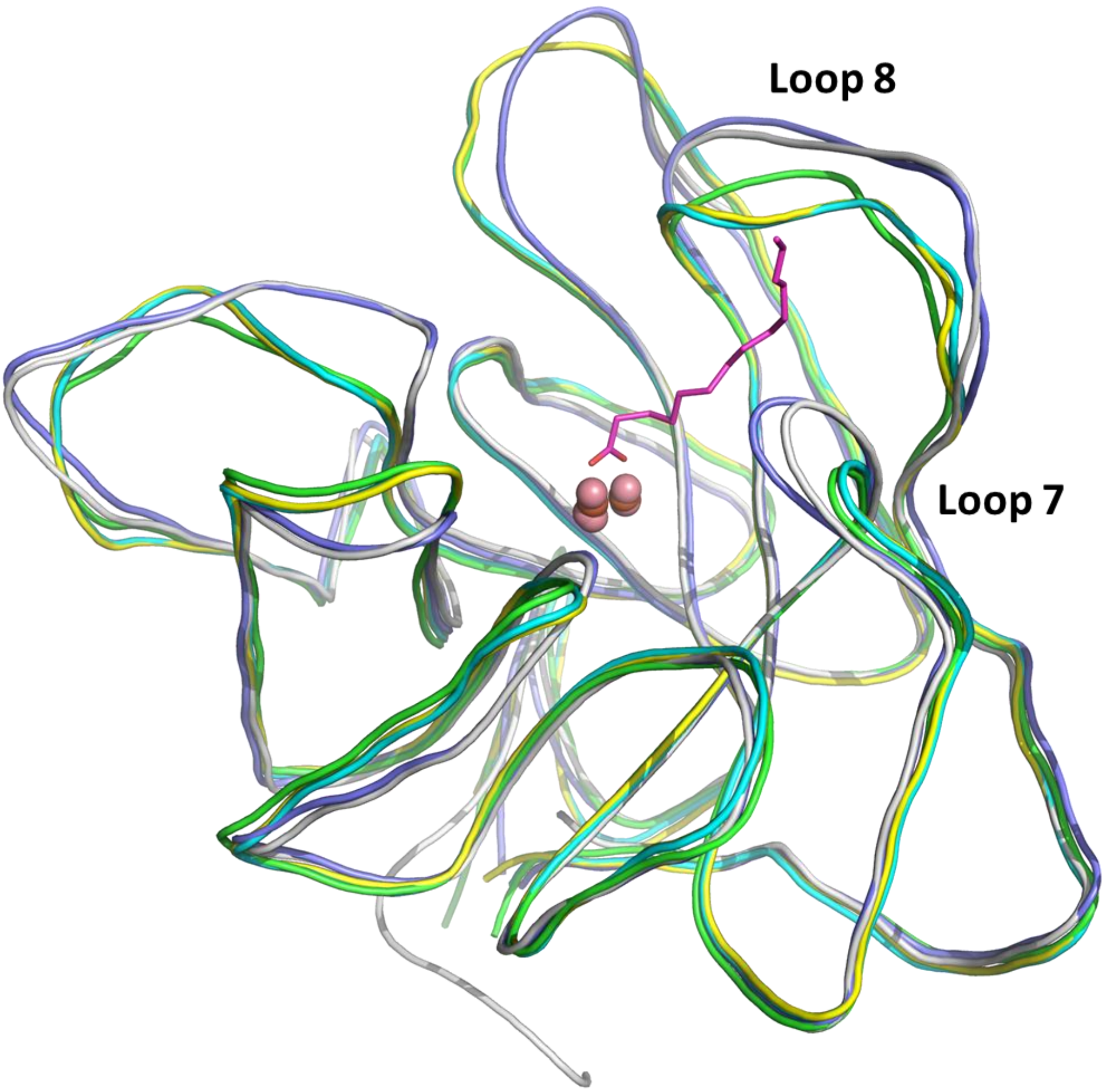

<u>Figure S3:</u> <u>Structural superposition of several PLLs with *Vmo*Lac</u>

Structures of *Vmo*Lac (green), *Sso*Pox (cyan, 2VC5), *Sis*Lac (yellow, 4G2D), *Dr*OPH (grey, 2ZC1) and *Gk*L (blue, 3OJG) are represented as smoothed ribbon. The bimetallic center is represented by spheres: light pink for cobalt, orange for iron and blue for zinc.

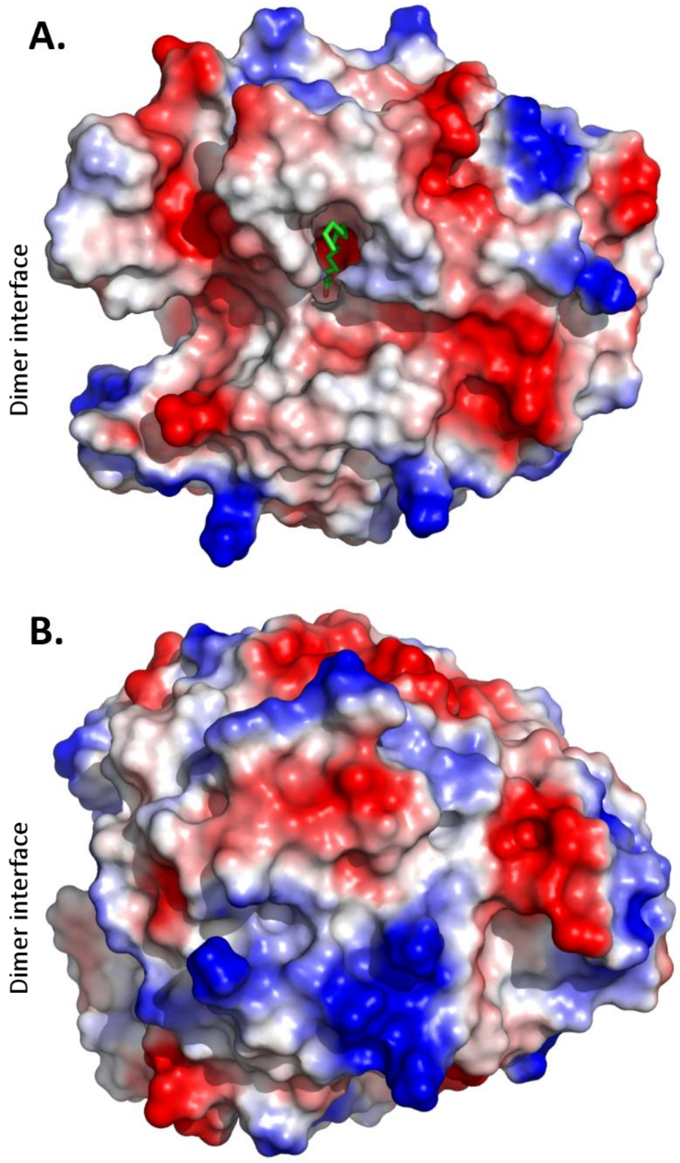

<u>Figure S4:</u> <u>Surface electrostatic potential of *Vmo*Lac</u>

The surface electrostatic potential of a monomer is represented on face (**A**) and face (**B**). Negatively charged areas are depicted in red, whereas positively charged areas are in blue and uncharged areas are in white. The active site crevice is indicated by the presence of the bound fatty acid (green sticks, face (**A)**)

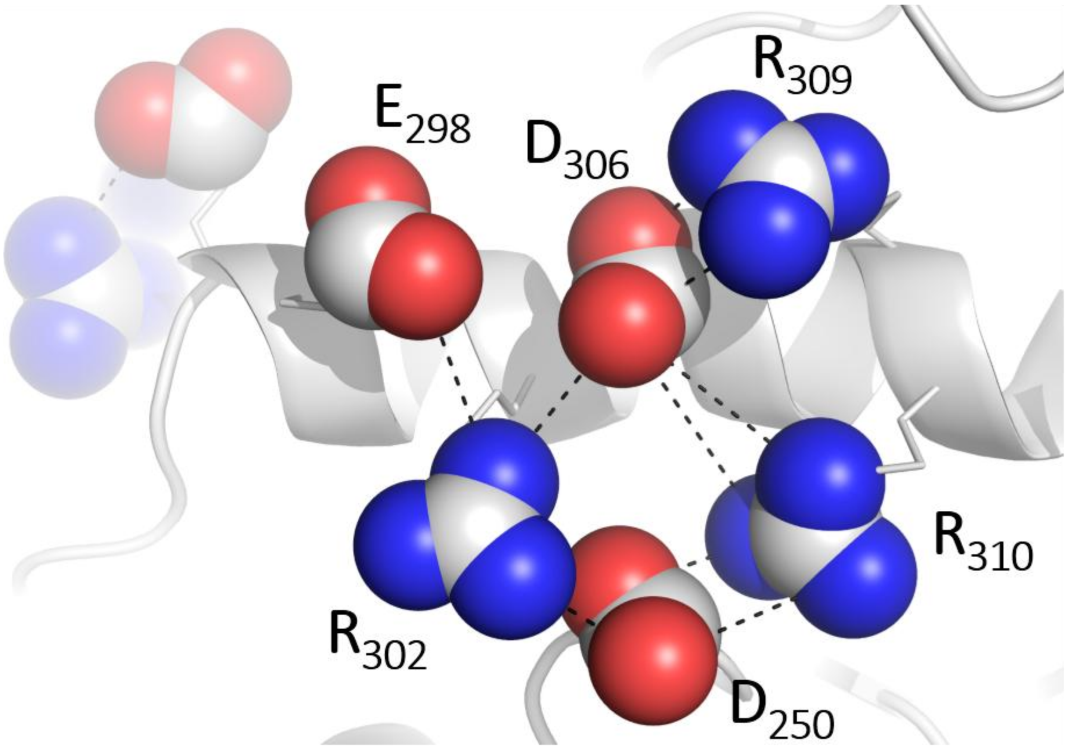

Figure S5: Surface salt bridge network example in the *Vmo*Lac structure

The charge groups of involved residues are shown as spheres. Ionic interactions are indicated by black dashes.

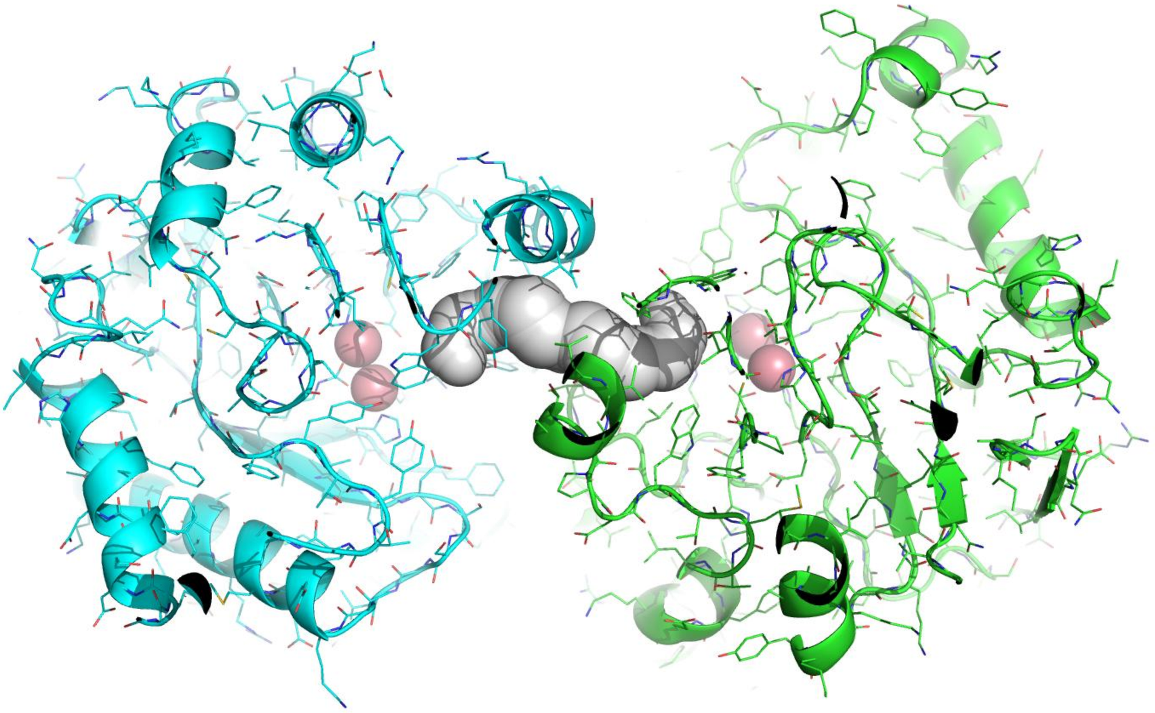

Figure S6: Contacts between *Vmo*Lac active site in the packing contact

*Vmo*Lac molecules are shown in cartoon (green and blue) at the crystallographic interface. The active site channels of both monomers (grey surface) are connected. The two active site metal

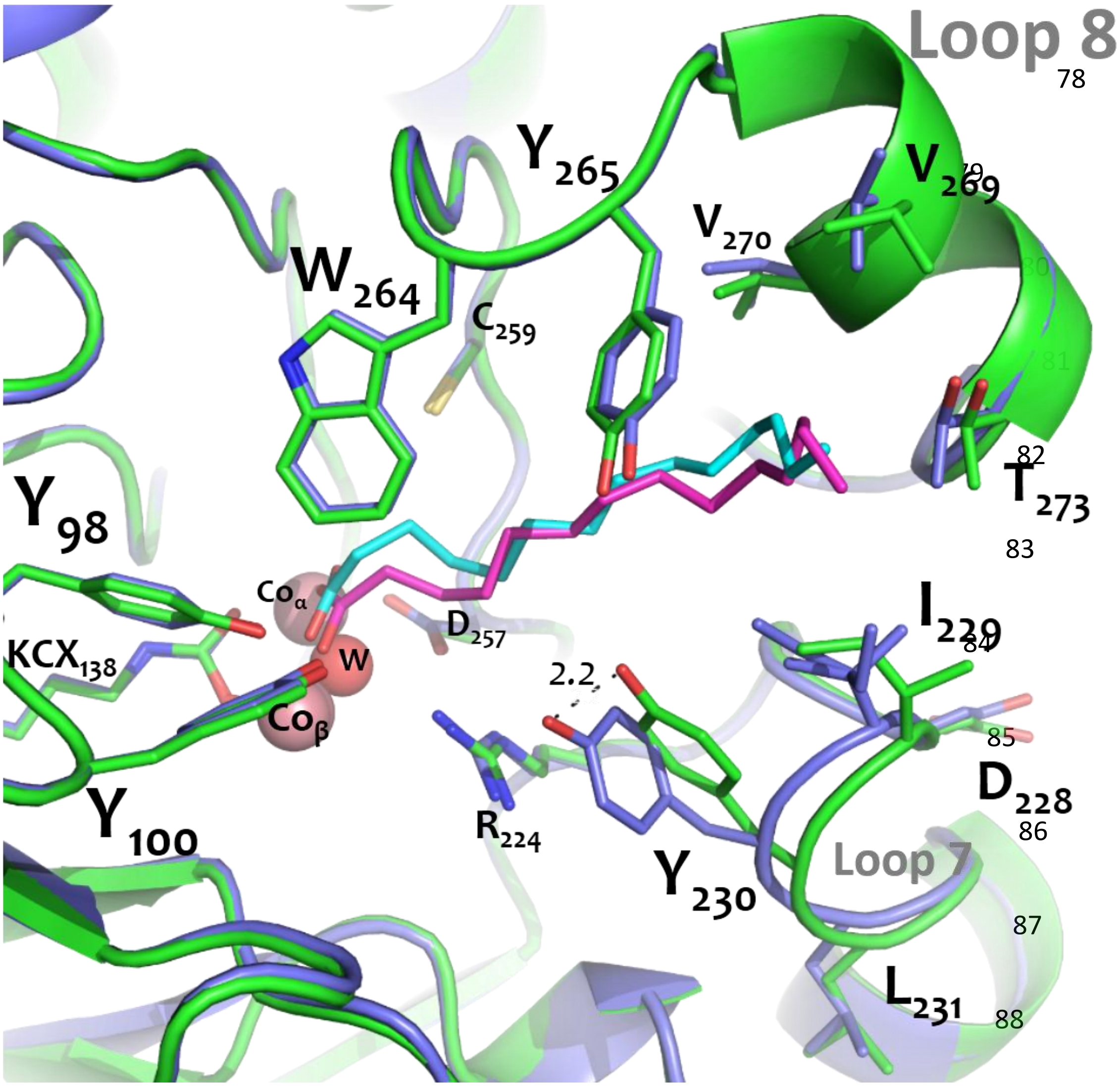

Figure S7: Superposition of *Vmo*Lac structures solved in $P6_4$ (green) and P622 space groups.

Active site superposition of *Vmo*Lac monomers from $P6_4$ (green) and P622 (dark blue) space group crystals. Colors are conserved as previously. Active site residues are represented as sticks.

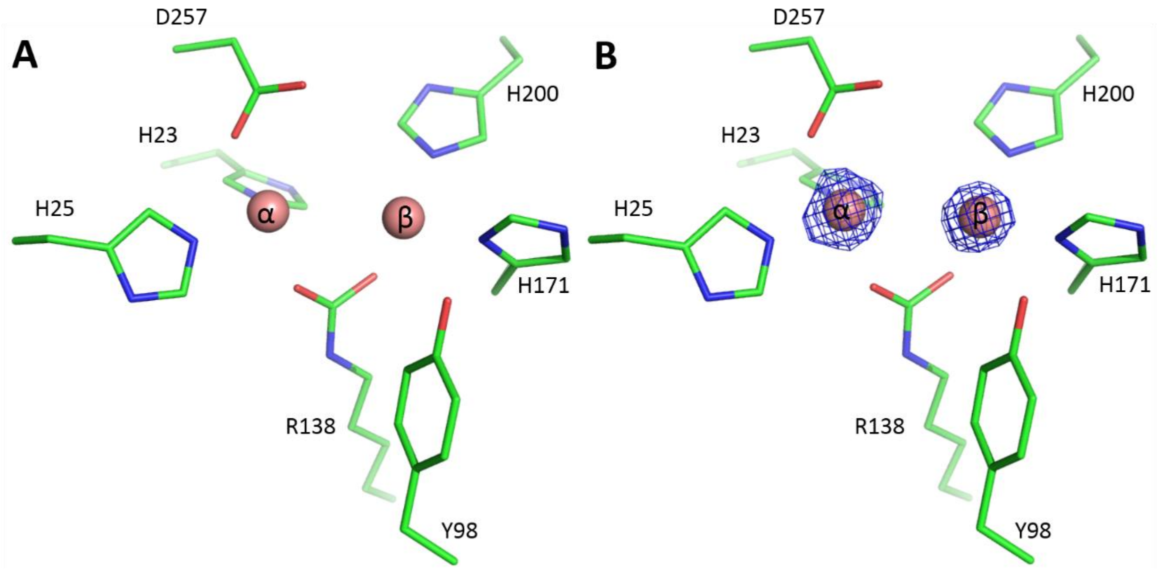

Figure S8: Anomalous X-ray scattering characterization of the *Vmo*Lac bimetallic center.

The Bijvoet difference Fourier maps from data collections at the arsenic atom peak (energy = 7.725kEV (**B**)) or remote (7.7kEV (**A**)) are contoured at 5σ. The high anomalous peaks (14 and 11.5σ for α and β sites, respectively) at the cobalt atom anomalous edge and the absence of anomalous signal at slightly lower energy indicate the presence of cobalt in the protein's active site.

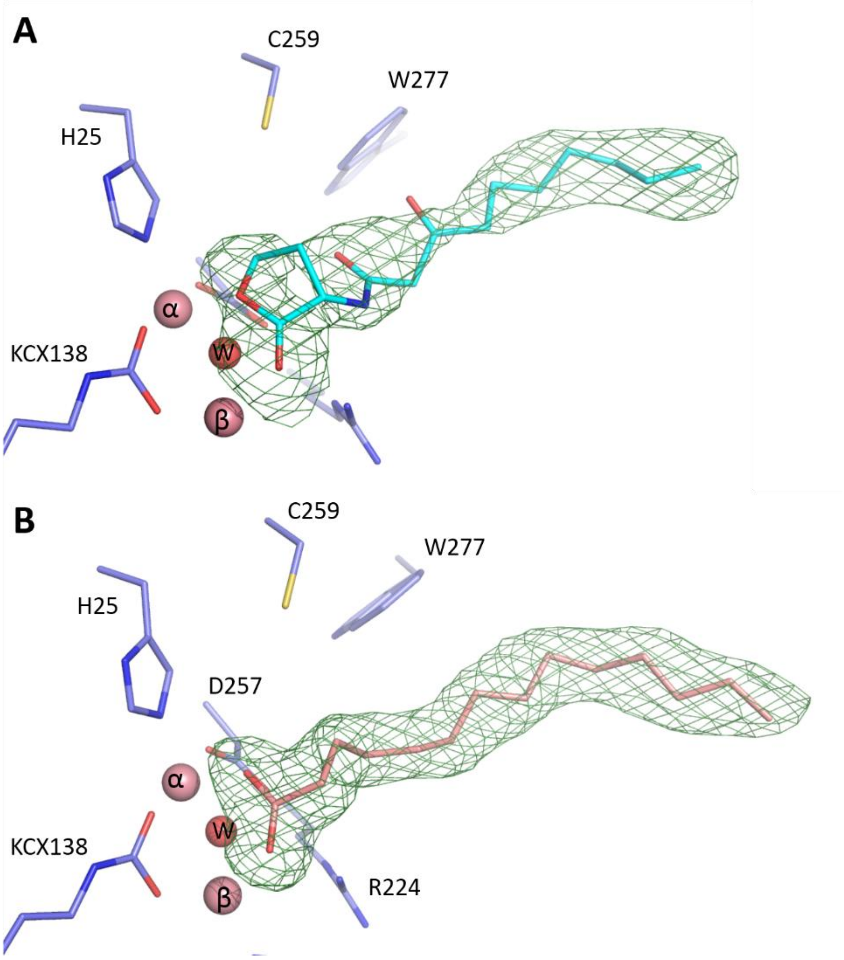

<u>Figure S9:</u> <u>Fourier difference omit maps of the bound ligands in *Vmo*Lac structures.</u>

(A) *Vmo*Lac structure (blue sticks) bound to 3-oxo-C10 AHL (cyan sticks). The omit F_{obs}-F_{calc} is contoured at 2.5 σ (green mesh). (B) *Vmo*Lac structure (blue sticks) bound to a fatty acid, modelled as myristic acid (pink sticks). The omit F_{obs}-F_{calc} is contoured at 3 σ (green mesh). Metal cations and the putative catalytic water molecule are shown as spheres (pink and red, respectively).

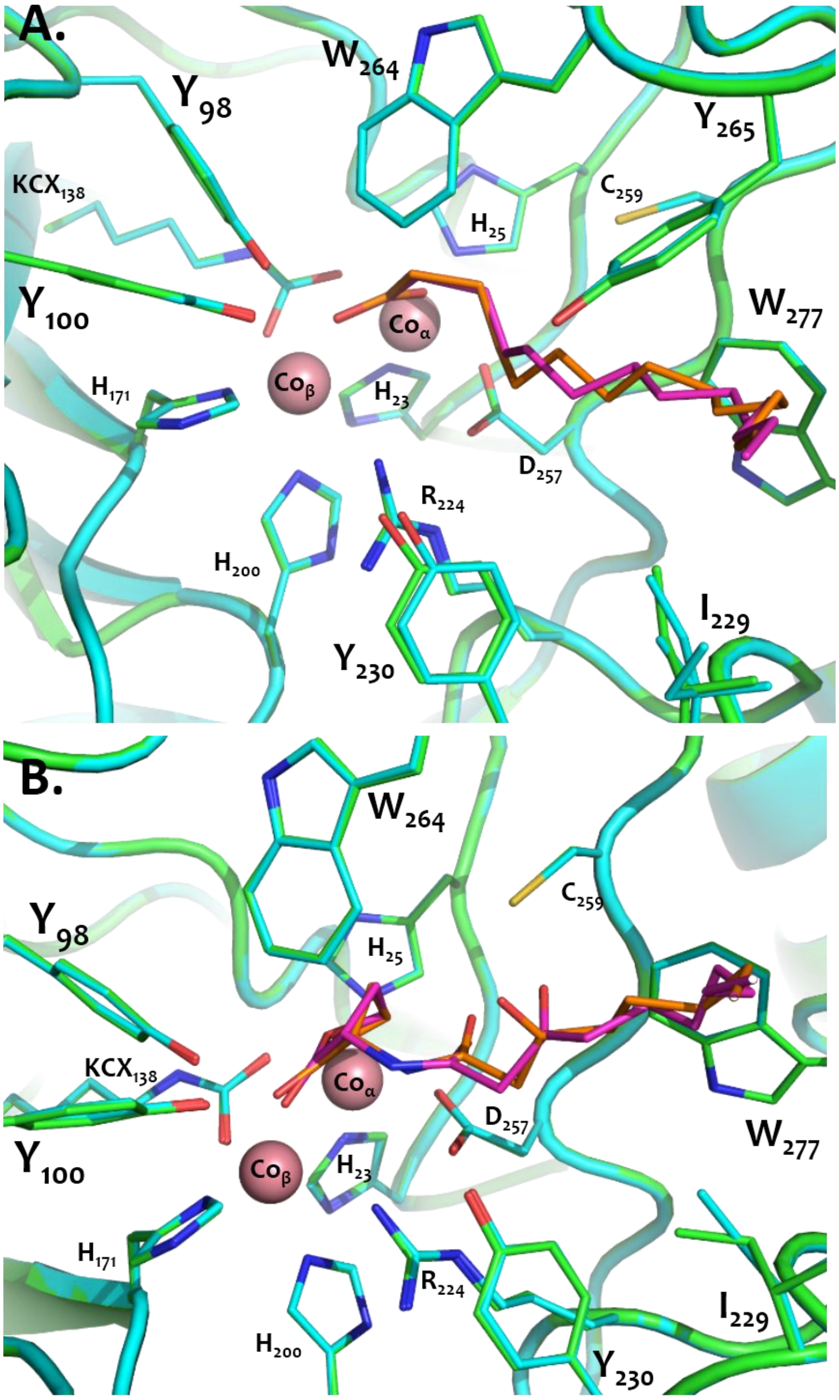

Figure S10: Superposition of monomers from homodimers of *Vmo*Lac bound to a fatty acid (**A.**) and to 3-oxo-C10 AHL (**B.**)

Monomers A and B for each of the two structures were superposed. Each monomers are represented in green and cyan with respective bound molecule in pink and orange. Cobalt atoms are represented as light pink balls and residues as sticks.

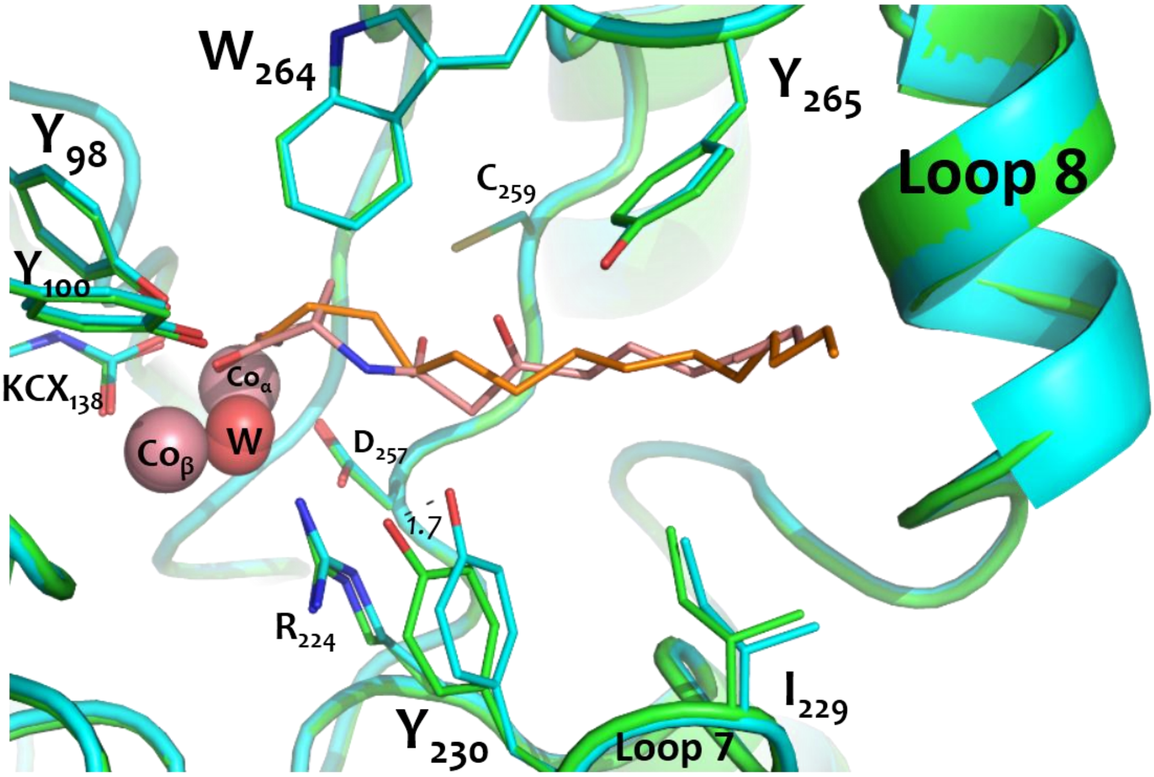

Figure S11: Superposition of the structure bound to a fatty acid with the structure bound to the AHL.

Comparison of the binding mode of the fatty acid (modeled as myristic acid (orange sticks) bount to *Vmo*Lac (green sticks and cartoon)) and 3-oxo-C10 AHL (light pink sticks, bound to *Vmo*Lac (cyan sticks and cartoon)). Cobalt cations and the bridging water molecule are represented by light pink and red spheres, respectively.

Table S1: Accession numbers of the sequences used in the phylogeny study

Sequence	NCBI Accession number
***Gs*P** (*Geobacillus thermodenitrificans*)	YP_001125472.1
***Gk*L** (*Geobacillus kaustophilus*)	YP_147359.1
***Dr*OPH** (*Deinococcus radiodurans*)	NP_294654.1
**1HZY PTE** (*Pseudomonas diminuta*)	GI:13786715
**PTEflavob** (*Flavobacterium* sp.)	AAV39527.1
**PTEAgrobac** (*Agrobacterium tumefaciens*)	AAK85308.1
**2R1M opd** (*Agrobacterium radibacter*)	GI:167744959
**PLLBreviba** (*Brevibacterium mcbrellneri*)	WP_005881372.1
**PLLDermaco** (*Dermacoccus* sp.)	WP_006945246.1
**AhlA** (*Rhodococcus erythropolis*)	WP_003943005.1
**QsdA** (*Rhodococcus erythropolis*)	ABQ42704.1
**PLLRhodoco** (*Rhodococcus jostii*)	YP_701486.1
**PLLStrepto** (*Streptosprangium roseum*)	YP_003338238.1
**MCP** (*Mycobacterium avium* subsp. *paratuberculosis* K-10)	NP_962602.1
**PPH** (*Mycobacterium tuberculosis*)	NP_214744.1
**PLLMycobCD** (*Mycobacterium tuberculosis*)	NP_214744.1
**PLLMycbovi** (*Mycobacterium bovis*)	NP_853900.1
SacPox (*Sulfolobus acidocaldarius*)	YP_256726.1
***Sis*Lac** (*Sulfolobus islandicus*)	YP_002828495.1
***Sso*Pox** (*Sulfolobus solfataricus*)	NP_343863.1
***Vmo*Lac** (*Vulcanisaeta moutnovskia*)	YP_004245953
**Symbact** (*Symbiobacterium thermophilum*)	YP_074383.1
**PHP_E** (*Escherichia coli*)	YP_001723339.1
**PHP_P** (*Photorhabdus asymbitoca*)	YP_003041416.1
**PHP_Y** (*Yersinia aldovae*)	WP_004702383.1
**PHP_X** (*Xenorhabdus bovienii*)	YP_003466084.1
**RTX_K1** (*Klebsellia pneumoniae*)	YP_001335395.1
**RTX_K2** (*Klebsellia variicola*)	YP_003439523.1
**RTX_P** (*Pseudomonas syringiae*)	EGH93047.1
**RTX_3K** (*Rhodobacter sphaeroides*)	YP_001045290.1

Table S2: Protein sequence identity between PLLs and *Bd*PTE

	***Bd*PTE**	***Sso*Pox**	***Sis*Lac**	***Sac*Pox**	***Vmo*Lac**	**PPH**	**MCP**	**AhlA/QsdA**	***Dr*OPH**	***Gk*L**	***Gs*P**
***Bd*PTE**	-	32.8	32.2	33.8	29	34.8	35.7	28.9	30.34	27.2	27.2
***Sso*Pox**		-	91.4	76.1	52	39.2	37.6	37.9	28.34	33.8	32.9
***Sis*Lac**			-	76.1	51.27	37.6	36.31	36.6	27.4	33.8	32.2
***Sac*Pox**				-	51.9	39.8	39.2	38.5	30.6	33.1	32.2
***Vmo*Lac**					-	41.1	40.8	36	29	29.6	28.3
**PPH**						-	92	59	31.6	32.7	32.4
**MCP**							-	58.7	31.6	32.7	32.7
**AhlA/QsdA**								-	32	30.8	31.1
***Dr*OPH**									-	58.8	60.7
***Gk*L**										-	90.4

Table S3: Anomalous X-ray data collection

Data collection		
Dataset	Co-K edge high	Co-K edge low
Beamline	ID29	ID29
Wavelength (Å)	1.6049 Å	1.6101 Å
Detector	PILATUS 6M	PILATUS 6M
Oscillation (°)	0.1	0.1
Number of frames	3600	3600
Resolution (Å) (last bin)	1.8 (1.9-1.8)	1.7 (1.8-1.7)
Space group	$P6_4$	$P6_4$
Unit-cell parameters (Å)	a = 174.87, b = 174.87, c = 62.07, α = 90, β = 90, γ = 120	a = 174.85, b = 174.85, c = 62.06, α = 90, β = 90, γ = 120
No. of observed reflections (last bin)	1 940 202 (258 680)	2 242 020 (321 007)
No. of unique reflections (last bin)	196 781 (29 433)	232 940 (36 350)
Completeness (%)(last bin)	100 (100)	99.8 (98.7)
R_{meas} (%) (last bin)	7.3 (53.2)	9.5 (74.1)
I/σ(I) (last bin)	21.74 (4.12)	16.25 (2.89)
Redundancy (last bin)	9.86 (8.79)	9.62 (8.83)
CC (1/2) (%)(last bin)	99.9 (90.9)	99.9 (81.3)
Anomalous peak (σ)[#]	α metal = 14<br>β metal = 11.5	N/D
[#] is the height of the anomalous peak (in σ units) in the Bijvoet difference Fourier map at the location of the metal cations in the structure. The anomalous signal decline at lower energy, showing that the binding pocket is occupied by zinc ions, but not only. N/D is for not detectable.		
